# High Sensitivity Bi_2_O_3_/Ti_3_C_2_T_x_ Ammonia Sensor Based on Improved Synthetic MXene Method at Room Temperature

**DOI:** 10.3390/s24206514

**Published:** 2024-10-10

**Authors:** Baocang Zhou, Zhihua Zhao, Zhenli Lv, Zhuo Chen, Sibo Kang

**Affiliations:** College of Mechanical and Electrical Engineering, Henan University of Technology, Zhengzhou 450052, China; keaiduo_2023@haut.edu.cn (B.Z.); lvzhenli@stu.haut.edu.cn (Z.L.); zhuo.chen@haut.edu.cn (Z.C.); 221020800311@stu.haut.edu.cn (S.K.)

**Keywords:** ammonia sensor, Bi_2_O_3_, Ti_3_C_2_T_x_, MXene, gas sensors

## Abstract

The MXene Ti_3_C_2_T_x_ was synthesized using hydrofluoric acid and an improved multilayer method in this study. Subsequently, a Bi_2_O_3_/Ti_3_C2T_x_ composite material was produced through hydrothermal synthesis. This composite boasts a unique layered structure, offering a large surface area that provides numerous contact and reaction sites, facilitating the adsorption of ammonia on its surface. The prepared Bi_2_O_3_/Ti_3_C_2_T_x_-based sensor exhibits excellent sensing performance for ammonia gas, including high responsiveness, good repeatability, and rapid response–recovery time. The sensor’s response to 100 ppm ammonia gas is 61%, which is 11.3 times and 1.6 times the response values of the Ti_3_C_2_T_x_ gas sensor and Bi_2_O_3_ gas sensor, with response/recovery times of 61 s/164 s at room temperature, respectively. Additionally, the gas sensitivity mechanism of the Bi_2_O_3_/Ti_3_C_2_T_x_-based sensor was analyzed, and the gas sensing response mechanism was proposed. This study shows that the sensor can effectively enhance the accuracy and precision of ammonia detection at room temperature and has a wide range of application scenarios.

## 1. Introduction

Ammonia, a colorless gas with a strong, pungent odor at room temperature, finds extensive use in the electronics, food, and chemical industries, as well as in scientific research. When released into the atmosphere, ammonia reacts with nitrogen and sulfur oxides, contributing to smog formation, which reduces visibility and poses severe health risks, particularly to the respiratory and cardiovascular systems [1]. Prolonged exposure to low concentrations of ammonia can cause significant health issues, including burns to the eyes and skin, respiratory damage, and even death [2,3]. Additionally, ammonia is a natural metabolic byproduct in the human body. Elevated levels of ammonia in exhaled breath can indicate liver, kidney, or lung diseases [4,5]. For instance, patients with end-stage renal disease (ESRD) often have an average exhaled ammonia concentration exceeding 4.88 ppm [6]. Therefore, developing gas-sensitive materials with large specific surface areas and high carrier mobility [7,8] for precise ammonia detection at room temperature is crucial for health and safety.

Various materials have been developed for ammonia detection, including metal oxides, conductive polymers, and carbon-based materials [9,10,11,12]. Metal oxides like zinc oxide, copper oxide, and tin oxide are commonly used due to their good response, sensitivity, and selectivity. For example, Bhardwaj et al. tested SnO_2_, and Meng et al. tested TiO_2_ for gas sensitivity [2,3]. Although these materials show good gas sensitivity, they require high operating temperatures, making precise room temperature detection challenging. Hence, there is a need to develop high-performance ammonia sensors capable of accurate detection at room temperature.

Metal oxide semiconductors are gaining attention for their excellent bandgap, high electron mobility, and two-dimensional structure and are increasingly used in gas-sensitive material development [13,14]. MXene, a two-dimensional compound composed of transition metal carbides, carbonitrides, or nitrides, has a high specific surface area, abundant active sites, and excellent conductivity, making it promising for gas sensor applications [15,16,17]. Discovered by Naguib et al. in 2011 [18], MXene has since been widely researched and applied in ammonia detection. Lu et al. successfully prepared a MXene/Na_2_Ti_3_O_7_ @PANI-composite-based gas sensor that is highly sensitive (185.44%) to 100 ppm NH_3_ at 45% RH, and a relative response of 283.8% was obtained at 90% RH along with a low detection limit of 186 ppb [19]. Wang et al. prepared a Ti_3_C_2_ MXene multilayer and TiO_2_ using a novel preparation technique based on the liquid-phase deposition of (NH_4_)_2_TiF_6_. The UOFEs coated with Ti_3_C_2_ MXene/TiO_2_ hybrid films were 12-fold more sensitive to RI with a maximum RI response value of 943–1056%/RIU than bare fibers [20]. Yu et al. successfully obtained superior NH_3_ sensors with high response and stability by wrapping the SnO_2_ nanoparticles on Ti_3_C_2_T_x_ MXene nanosheets via a facile hydrothermal method, whose observed response, rapid response, and recovery times were 109, 342 s, and 75% for 500 ppm NH_3_ gas, respectively [21]. Hou et al. prepared a MXene Ti_3_C_2_T_x_-TiO_2_-CuO by one-step in situ oxidation method using Cu(NO_3_)_2_•6H_2_O as a precursor, which exhibited an improved NH_3_ gas sensing response of 56.9 toward 100 ppm NH_3_ under ultraviolet (UV) light exposure at room temperature [22]. To enhance sensor performance, heterostructured materials with unique physical and chemical properties have become a research focus in nanomaterials and composite materials [23]. Bi_2_O_3_, with its large bandgap and surface area [24], is effective in detecting organic compounds, metal ions, humidity, and gases [25,26,27,28,29]. However, there are no reports on the ammonia detection performance of Bi_2_O_3_-modified Ti_3_C_2_T_x_ MXene materials.

In this study, MXene Ti_3_C_2_T_x_ was synthesized using hydrofluoric acid and an improved multilayer method, and Bi_2_O_3_/Ti_3_C_2_T_x_ nanocomposites were synthesized via hydrothermal methods. The material’s composition, crystalline structure, and appearance were analyzed using XRD, SEM, and XPS techniques. The results indicate that Bi_2_O_3_/Ti_3_C_2_T_x_ nanocomposites exhibit high response values to ammonia gas, good repeatability, and rapid response and recovery times, making them promising for ammonia detection applications in agricultural, industrial, medical, health care, and other fields.

## 2. Experiments

Ti_3_C_2_T_x_ was prepared by removing the Al layer of the MAX phase (Jilin 11 Technology Co., Ltd., Jilin, China). Bismuth nitrate pentahydrate (AR, 99%) was purchased from McLean Biochemical (Shanghai Technology Co., Ltd., Shanghai, China). HF aqueous solution (AR, ≥40%), Ammonia aqueous (AR), and Sodium Hydroxide (ACS, K ≤ 0.02%, ≥97.0% (T), Falkes) were obtained from Shanghai Aladdin Biochemical Technology Co., Ltd., Shanghai, China. Anhydrous ethanol, formaldehyde, acetone, dimethylformamide, methanol, and glacial acetic acid were purchased from Sinopharm Chemical Reagent Co., Ltd., Shanghai, China.

### 2.1. Synthesis of Multilayer Ti_3_C_2_T_x_ MXene

Using the improved method from Alhabeb et al. [30], the multilayer Ti_3_C_2_T_x_ was prepared. To achieve this, the desired multilayer MXene was produced by etching Ti_3_AlC_2_ with HF. A total of 1 g of MAX phase material was slowly (over 10–15 min) added to 20 mL of hydrofluoric acid. The mixture was stirred continuously for 24 h with a magnetic stirrer at 500 r/min and 35 °C. After stirring, the resulting black suspension was collected and centrifuged at 3500 r/min with deionized water. This centrifugation was repeated until the supernatant reached a pH value close to 6. The supernatant was then separated, and the sediment was collected. Then, the sediment was dried under vacuum at 60 °C for 12 h, producing multilayer Ti_3_C_2_T_x_ MXene.

### 2.2. Synthesis of Bi_2_O_3_/Ti_3_C_2_T_x_ Nanocomposites

**Figure 1:**
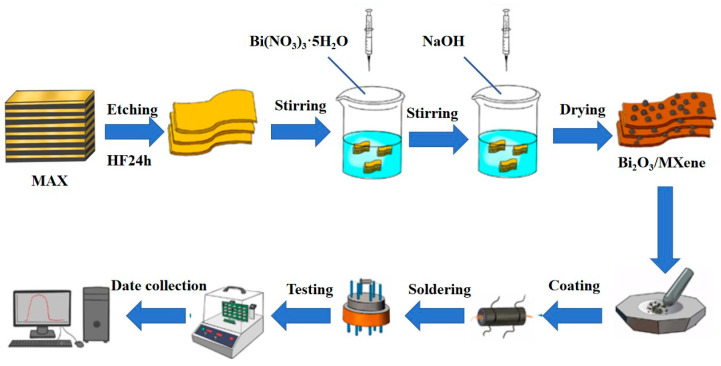
A flowchart of the experimental procedure.

The Bi_2_O_3_/Ti_3_C_2_T_x_ nanocomposites were synthesized using a hydrothermal method. 500 mg of Bi(NO_3_)_3_•5H_2_O was accurately measured and mixed with 10 mL of diluted nitric acid solution, ensuring complete dissolution through stirring. A total of 100 mg of multilayer Ti_3_C_2_T_x_ was then gradually added to this solution while maintaining stirring for over 30 min to ensure complete integration. This mixture was subsequently added to a 5 mol/L NaOH solution and stirred at 500 r/min and 80 °C for 30 min. The resulting precipitate was collected by centrifugation and dried under vacuum at 60 °C for 12 h to obtain Bi_2_O_3_/Ti_3_C_2_T_x_ nanocomposites. Different loading ratios were prepared using the same method, and the products were labeled BO/M-4, BO/M-5, and BO/M-6 for differentiation. Pure Bi_2_O_3_ was prepared using the same method without adding multilayer Ti_3_C_2_T_x_. Figure 1 illustrates the detailed experimental procedure.

### 2.3. Material Characterization

The crystal structures, valency, and microscopic morphology of the as-prepared samples were investigated using X-ray diffraction (XRD, X’Pert PRO, Malvern Panalytical Ltd., Almelo, The Netherlands) with Cu Kα radiation (λ = 1.5442 Å), X-ray photoelectric spectroscopy (XPS, PHI-5300, Perkin Elmer, Waltham, MA, USA) with a monochromatic Al Kα radiation (1486.6 eV), and scanning electron microscope (SEM, Gemini SEM 300, ZEISS, Oberkochen, Germany), respectively. During the analysis process, the XRD detection parameters are set as a current density of 40 mA, a tube voltage of 40 kV, with the scan angle ranging from 5° to 90°, and a scan rate of 10° per minute.

### 2.4. Fabrication and Measurement of Gas Sensor

A total of 20 mg of Bi_2_O_3_/Ti_3_C_2_T_x_ nanocomposites was added to an appropriate amount of absolute ethanol, and the mixture was sonicated for 2 min until the composite material was sufficiently dissolved. Then, it was uniformly coated in thickness to Al*_2_*O*_3_* ceramic tubes with Au electrodes. The dried ceramic tube, which had been vacuum dried at 80 °C for 6 h, was welded to the base correspondingly. After that, the prepared sensor was aged for 6 h using a WS-30B gas-sensitive element test system (Zhengzhou Wensen Electronic Technology Co., Ltd., Zhengzhou, China) to ensure its stability and reliability. As shown in Figure 1, the gas-sensitive performance experiments of the aged sensor were performed at room temperature (25 °C) by WS-30B. Additionally, pure Bi_2_O_3_-based and Ti_3_C_2_T_x_-based sensors were tested using the same methodology for comparison, respectively.

The gas-sensitive response of the sensors to common hazardous gases, including NH_3_, C_2_H_5_OH, CH_3_OH, DMF, CH_3_COOH, HCHO, and CH_3_COCH_3_, was tested, respectively, at 25 °C. After obtaining a stable baseline, the tested gases were injected into the WS-30B chamber with a microsyringe, and the evaporation was accelerated by heating. After a period of adsorption, a stable baseline was obtained again. As a typical N-type semiconductor, the response value (*R*_s_) of the Bi_2_O_3_/Ti_3_C_2_T_x_-based sensor to the detected gas can be defined by Equation (1):

*R*_s_ = |*R*_a_ − *R*_g_|/*R*_a_ × 100% (1)

where *R*_a_ and *R*_g_ denote the resistance of the sensor in pure air versus in a detected gas environment, respectively.

## 3. Results

### 3.1. Characterization Results

**Figure 2:**
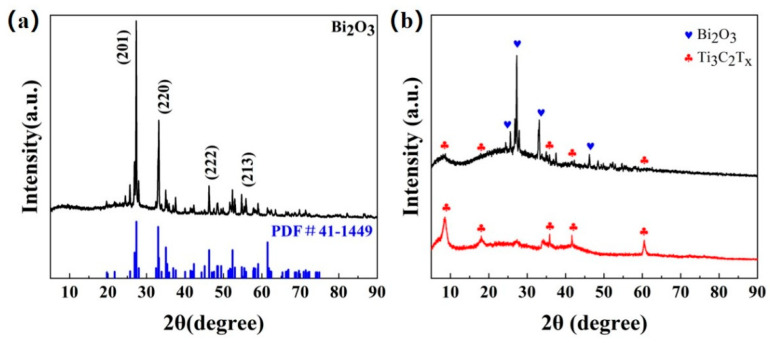
(**a**) XRD patterns of Bi_2_O_3_. (**b**) XRD patterns of Ti_3_C_2_T_x_ MXene and Bi_2_O_3_/Ti_3_C_2_T_x_ nanocomposites.

The XRD patterns of Bi_2_O_3_, Ti_3_C_2_T_x_ MXene, and Bi_2_O_3_/Ti_3_C_2_T_x_ are shown in Figure 2a and Figure 2b, respectively. It can be observed that the diffraction peaks of the Bi_2_O_3_-based sample almost coincide with the (201), (220), (400), (222), and (213) facets of the standard XRD pattern of Bi_2_O_3_ (PDF card 41-1449), which indicates that body-centered cubic Bi_2_O_3_-based materials have been successfully prepared in this paper. For the XRD pattern of Ti_3_C_2_T_x_ MXene, the diffraction peaks at 9.0°, 18.6°, 27.5°, and 60.7° on the (002), (004), (006), and (110) facets were clearly observed, as shown in Figure 2b, which was consistent with the results observed by Kuang et al. [31]. Moreover, the characteristic peaks of Bi_2_O_3_ and Ti_3_C_2_T_x_ MXene can be observed in the XRD pattern of Bi_2_O_3_/Ti_3_C_2_T_x_ nanocomposites, which indicates that all of the materials have been successfully prepared.

**Figure 3:**
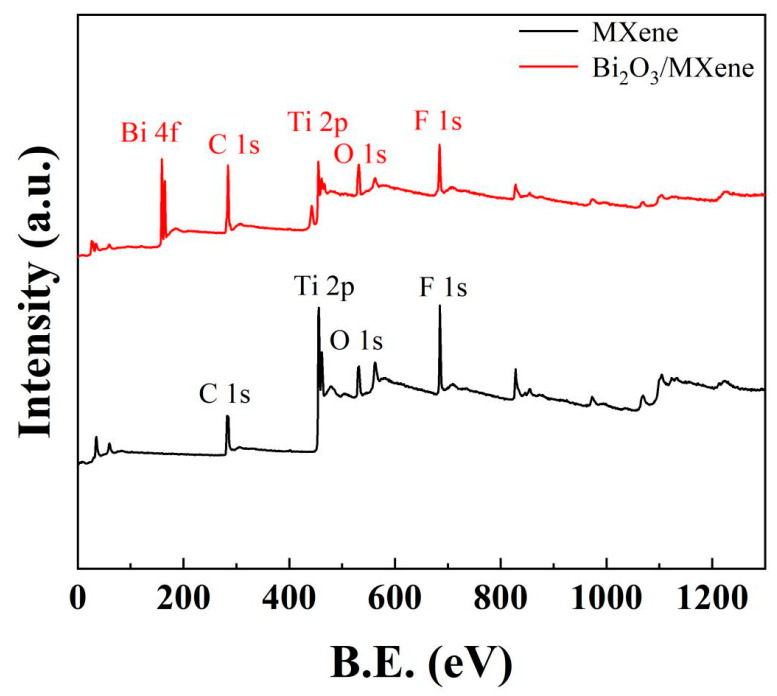
Full survey XPS spectrum of Ti_3_C_2_T_x_ MXene and Bi_2_O_3_/Ti_3_C_2_T_x_ nanocomposites.

Figure 3 represents the full survey XPS spectrum of Ti_3_C_2_T_x_ MXene and Bi_2_O_3_/Ti_3_C_2_T_x_ nanocomposites. It can be identified multiple elements in the full survey XPS spectrum of Ti_3_C_2_T_x_ MXene, such as C 1s, Ti 2p, O 1s, and F 1s. Meanwhile, the elemental mapping of Al in the MAX phase is not found, indicating that the present paper has successfully etched multilayer Ti_3_C_2_T_x_ MXene. Furthermore, the presence of elements O and F also indicates that the multilayer MXene surface was covered by -O and -F functional groups. The elemental Bi in Bi_2_O_3_ was also observed for C1s, Ti 2p, O 1s, and F 1s contained in multilayer Ti_3_C_2_T_x_ MXene shown in Bi_2_O_3_/Ti_3_C_2_T_x_ full pattern, especially the peak intensity of O element increases significantly because of the introduction of O element in Bi_2_O_3_. All of these proved that the Bi_2_O_3_/Ti_3_C_2_T_x_ nanocomposites have been successfully synthesized.

**Figure 4:**
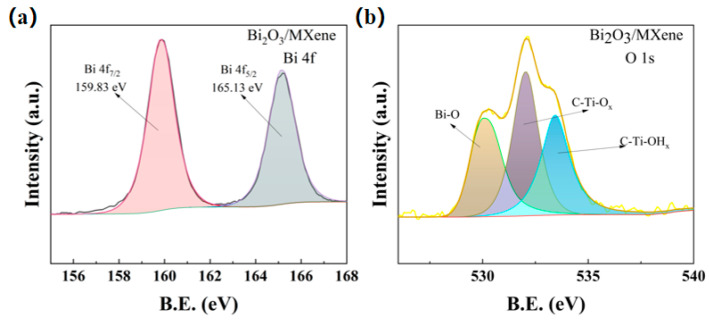
(**a**) XPS spectra of O 1s and (**b**) XPS spectra of Bi 4f for Bi_2_O_3_/Ti_3_C_2_T_x_ nanocomposites.

The C-Ti-OH_x_ can be observed from the XPS spectra of O1s in Figure 4a, which indicated that besides -O and -F functional groups, -OH functional groups also existed in the multilayer MXene. The XPS spectra of the 4f orbitals of Bi elements are shown in Figure 4b, and the spectral positions of the characteristic peaks of 159.83 eV and 165.13 eV for Bi 4f_7/2_ and Bi 4f_5/2_ indicate that the electronic binding energy difference between them is 5.3 eV, which are approximately equal to the positions of the spectral peaks of Bi^3+^ 3d in the standard XPS spectrograms. It demonstrates that the elemental Bi exists in the form of Bi^3+^ in the Bi_2_O_3_/Ti_3_C_2_T_x_ nanocomposites.

**Figure 5:**
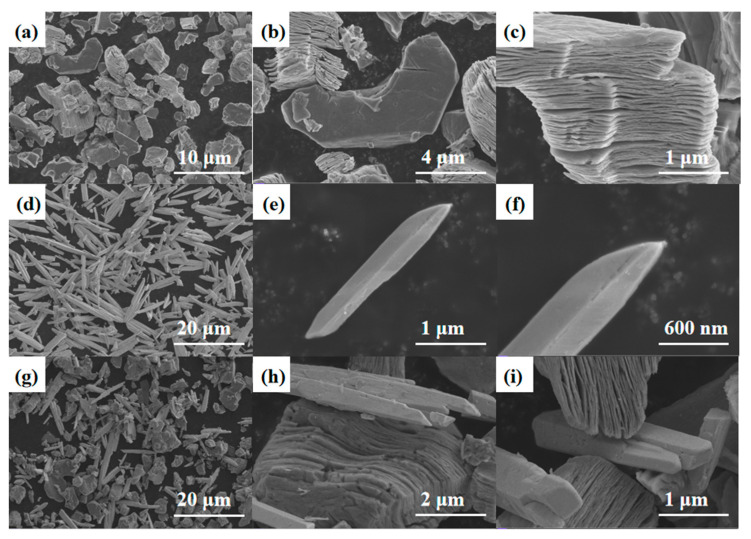
(**a**–**c**) SEM images of Ti_3_C_2_T_x_ MXene. (**d**–**f**) Pure Bi_2_O_3_ needle. (**g**–**i**) SEM images of Bi_2_O_3_/Ti_3_C_2_T_x_ nanocomposites.

Figure 5a–c show the SEM image of Ti_3_C_2_T_x_ MXene, where it can be observed that the MXene exhibits a multilayer structure similar to an accordion, and the surface of individual granular-like structures is smooth and free of impurities. SEM images of Bi_2_O_3_ are shown in Figure 5d–f, from which it can be observed that pure Bi_2_O_3_ exhibits a uniformly distributed needle structure. The SEM image of Bi_2_O_3_/Ti_3_C_2_T_x_ nanocomposites is shown in Figure 5g–i, from which it can be observed that Ti_3_C_2_T_x_ multilayer MXene and Bi_2_O_3_ are evenly distributed. The successfully prepared composite material has a uniformly distributed nanostructure, which makes it an excellent potential for NH_3_-sensitive sensing.

**Figure 6:**
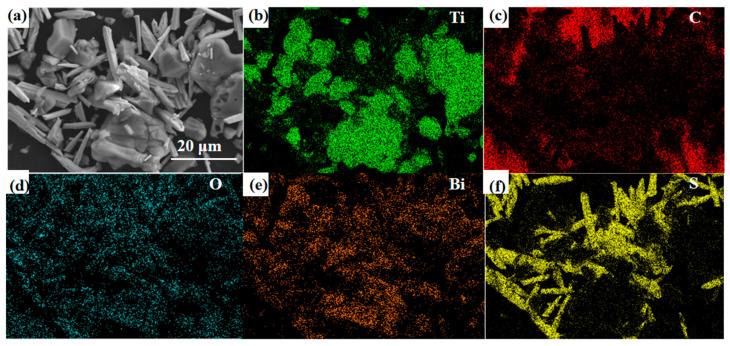
(**a**) SEM images and (**b**–**f**) EDS mappings of Bi_2_O_3_/Ti_3_C_2_T_x_ nanocomposites.

As shown in Figure 6, the element distribution state was tested using an energy spectrometer integrated with the SEM. The Ti, C, O, F, and Bi elements can be observed in the composite material. In this context, the oxygen (O) element is partly derived from the surface -O and -OH functional groups, while the fluorine (F) element originates from the -F functional group on the surface of MXene. These findings align with previous XPS analysis results, mutually verifying the conclusions. The correspondence between the bismuth (Bi) and oxygen (O) elements further confirms the successful synthesis of Bi_2_O_3_.

### 3.2. Gas Sensing Performance

#### 3.2.1. Comparison of Gas Sensitivity Performance of Sensors with Different Ratios to Ammonia

**Figure 7:**
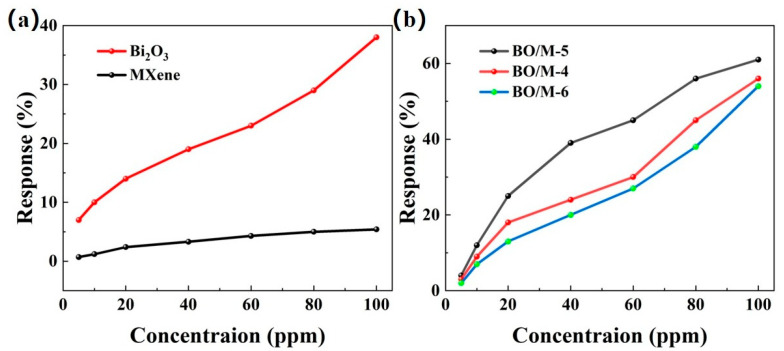
The response of pure Ti_3_C_2_T_x_, Bi_2_O_3_ sensors (**a**) and sensors BO/M-4, BO/M-5, and BO/M-6 with different composite ratios (**b**) to 100 ppm of ammonia at room temperature.

To evaluate the gas sensing performance of the prepared composite material sensors, a comparative experiment was conducted with pure Bi_2_O_3_ and Ti_3_C_2_T_x_ gas sensors to clarify their performance advantages. The response curves of different loading ratios to various concentrations of ammonia were tested at room temperature, and their response values were calculated. As shown in Figure 7, all the prepared gas sensors exhibited increasing response values with the increase in ammonia concentration. Figure 7a shows that the response values of pure Ti_3_C_2_T_x_ and pure Bi_2_O_3_ sensors from 5 ppm to 100 ppm ammonia were relatively lower than those of the Bi_2_O_3_ and Ti_3_C_2_T_x_ doped sensors, with pure Ti_3_C_2_T_x_ and Bi_2_O_3_ sensors showing responses of 5.4% and 38% at room temperature, respectively. Figure 7b shows that among the three different loading ratio sensors, the response value in the same testing environment was in the order of BO/M-5 > BO/M-4 > BO/M-6, with BO/M-5 (5:1) showing the highest response value, reaching 61% at room temperature. The BO/M-5 gas sensor’s response value was 11.3 times higher than that of Ti_3_C_2_T_x_ and 1.6 times higher than Bi_2_O_3_, demonstrating the effectiveness of doping. Hence, the BO/M-5 gas sensor is used for subsequent testing.

#### 3.2.2. Selectivity Test of BO/M-5 Gas Sensor

**Figure 8:**
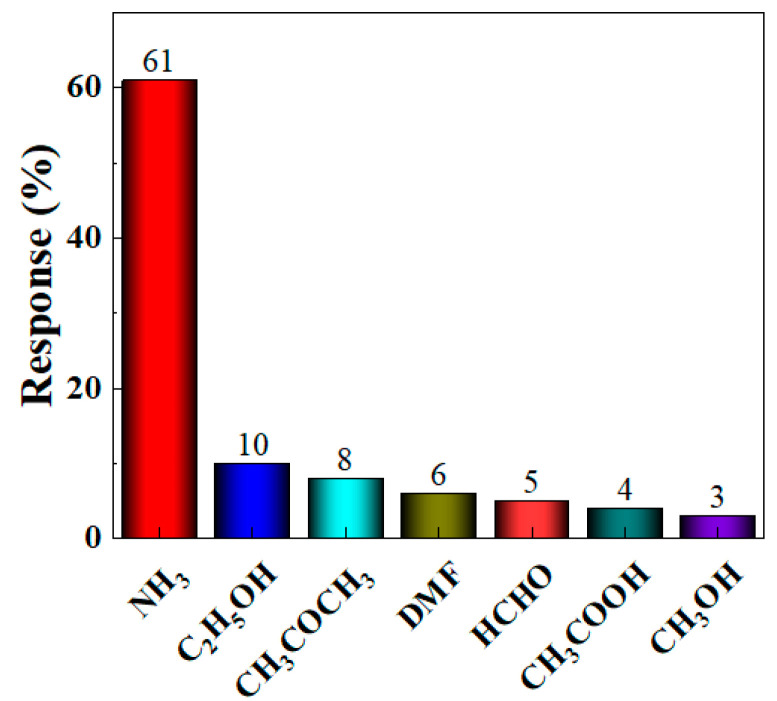
The response of the BO/M-5 gas sensor to detect 100 ppm of various gases.

Based on the optimal loading ratio, further in-depth research on the gas sensitivity characteristics of the BO/M-5 gas sensor with the optimal loading ratio is established. Selectivity measures the sensor’s ability to distinguish between different interfering gases at a specific concentration. To test the interference resistance of the BO/M-5 gas sensor, gases such as 100 ppm ammonia, acetone, ethanol, DMF, formaldehyde, acetic acid, and methanol were tested at room temperature, and their response values were calculated. Figure 8 shows that the BO/M-5 gas sensor had a significantly higher response to 100 ppm ammonia compared to other gases, demonstrating its excellent selectivity and sensitivity to ammonia (see Figure S1 in Supplementary Materials for detail). The sensor’s response to ammonia reached 61%, while responses to other gases were all below 10%. The response of the BO/M-5 sensor to ammonia was 6.1–20 times higher than to other gases, indicating excellent selectivity for ammonia.

#### 3.2.3. Gas Sensitivity Performance of BO/M-5 Gas Sensor at Different Humidities

**Figure 9:**
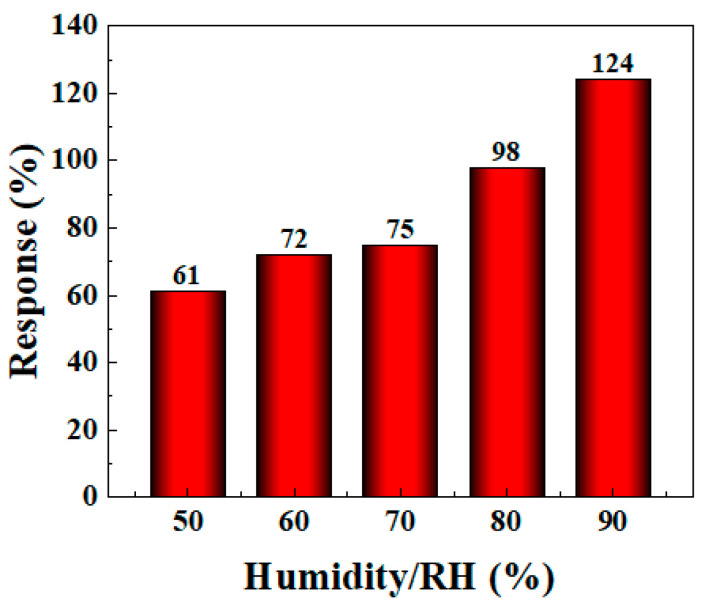
The response of BO/M-5 gas sensor to 100 ppm of ammonia at different levels of humidity.

The gas sensitivity performance of most gas sensors can be affected by different humidity conditions, and the detection environment often varies in practical applications. Therefore, it is very important to further study the sensor’s gas sensitivity performance under different humidity conditions. In the experiment, to simulate the effect of different humidity environments on the gas sensor’s performance, deionized water was added to the evaporation stage of the WS-30B gas sensor testing instrument and rapidly evaporated using a heating device to increase the humidity level in the test chamber. An external humidity meter was used to monitor the humidity in the closed chamber to achieve the target humidity. As shown in Figure 9, the gas sensitivity of the BO/M-5 sensor improved with increasing humidity (see Figure S2 in Supplementary Materials for detail). The response value reached 124% at 90% humidity, proving that the sensor can adapt to complex humidity environments and even exhibit enhanced gas sensitivity in high-humidity conditions, which is beneficial for ammonia detection in subsequent applications.

#### 3.2.4. Response Recovery Performance of the BO/M-5 Gas Sensor

**Figure 10:**
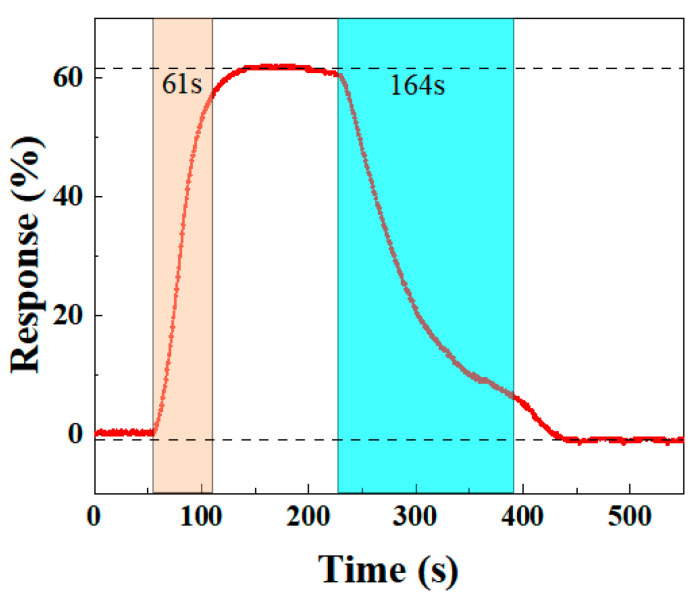
The response and recovery curves for the BO/M-5 gas sensor to 100 ppm ammonia.

An ideal gas sensor should have rapid response and recovery times to quickly detect the target gas and allow sufficient time for further processing. Thus, response and recovery times are crucial for evaluating sensor performance. Based on the definitions of Equation (1), the BO/M-5 sensor’s response time to 100 ppm ammonia is 61 s, while the recovery time is 164 s, as shown in Figure 10. The sensor’s resistance quickly drops upon NH_3_ injection for 55 s (red area in Figure 10), and after NH_3_ is removed at 230 s (blue area in Figure 10), the resistance starts to increase until it returns to its original state. These results show that the sensor performs well in both response and recovery times, supporting its potential for further applications.

#### 3.2.5. Repeatability and Long-Term Stability of BO/M-5 Gas Sensor

**Figure 11:**
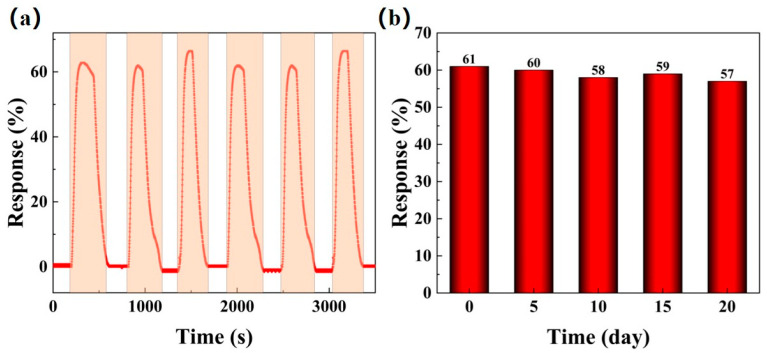
The repeatability (**a**) and long-term stability (**b**) of BO/M-5 to 100 ppm ammonia at room temperature under natural conditions.

For long-term use, gas sensors must exhibit good repeatability and stability to ensure consistent performance. Figure 11 investigates the repeatability and long-term stability of the BO/M-5 sensor with 100 ppm ammonia under natural conditions. When exposed repeatedly to 100 ppm ammonia at room temperature, the sensor showed consistent response values with minimal variation, as shown by the red line in Figure 11a. Additionally, the BO/M-5 sensor demonstrated excellent long-term stability in practical applications, with response values remaining stable over a 20-day period with tests conducted every five days, as shown in Figure 11b.

## 4. Discussion

The gas sensing mechanism of the sensor for ammonia was analyzed based on the sensing characteristics of the prepared materials. The resistance changes during the adsorption and desorption processes of the composite material indicate that the sensor acts as an N-type semiconductor with reduced resistance when ammonia molecules adsorb onto its surface. At room temperature, oxygen adsorbs on the composite material surface as O_2_^-^ [32]. When the sensor is exposed to ammonia, a redox reaction occurs, as shown in Equation (2):

4NH_3_ + 5O_2_^−^ → 4NO + 6H_2_O + 5e^−^ (2)

This reaction generates electrons, which further combine with hole carriers in Ti_3_C_2_T_x_, leading to an increase in resistance. Once the sensor is removed from NH_3_, the resistance gradually recovers to its initial or partially recovered state, as detailed in Equations (3) and (4):

2NH_3_ + 3O^−^ → N_2_ + 3H_2_O + 3e^−^ (3)

NH_3_ + OH^−^ → NH_2_ + H_2_O + e^−^ (4)

**Figure 12:**
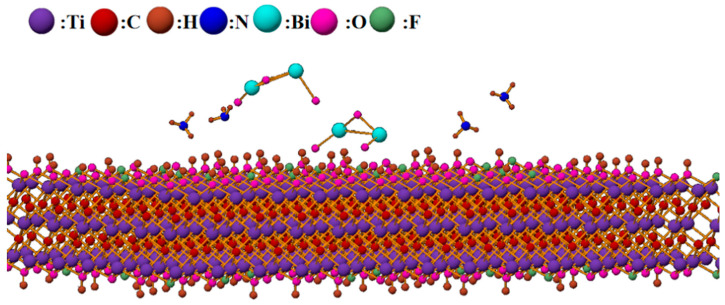
Schematic diagram of the reaction between Bi_2_O_3_/Ti_3_C_2_T_x_ nanocomposites and ammonia.

Furthermore, MXene materials possess high electronic mobility, which facilitates rapid carrier transport and enhances sensing performance, providing strong support for better sensing characteristics [5]. The large surface area of MXene provides more contact points and reaction sites for ammonia adsorption on the composite material surface. The surface of MXene also contains numerous functional groups and structural defects that play significant roles in adsorption and desorption. The gas sensing mechanism model of Bi_2_O_3_/Ti_3_C_2_T_x_ nanocomposites is shown in Figure 12.

## 5. Conclusions

In summary, Bi_2_O_3_/Ti_3_C_2_T_x_ nanocomposites were successfully prepared using the hydrothermal method. The materials were characterized using XRD, XPS, SEM, and EDS, and their gas-sensing properties were analyzed using WS-30B. XRD results confirmed that Bi_2_O_3_ matched the standard reference, and the XRD pattern of the prepared multilayer MXene was consistent with the literature data. Characteristic peaks for MXene and Bi_2_O_3_ were observed in the composite material. XPS analysis of composition and elemental valence states further confirmed the successful synthesis of the materials. The gas sensing performance of the prepared sensor was investigated with WS-30B. The Bi_2_O_3_/Ti_3_C_2_T_x_ sensor achieved a response value of 61% to 100 ppm ammonia at room temperature, outperforming Ti_3_C_2_T_x_ and Bi_2_O_3_ sensors by factors of 11.3 and 1.6, respectively. The sensor also demonstrated excellent repeatability and rapid response and recovery times (61 s/164 s). Moreover, the gas sensing mechanism of the Bi_2_O_3_/Ti_3_C_2_T_x_ sensor was also explored.

## Data Availability

Data are contained within the article.

## Supplementary Materials

The following supporting information can be downloaded at: https://www.mdpi.com/article/10.3390/s24206514/s1, Figure S1. The response of BO/M-5 sensor to detect 100 ppm of acetone, ethanol, DMF, formaldehyde, acetic acid and methanol gas. Figure S2. The response of BO/M-5 gas sensor to 100 ppm of ammonia at relative humidity 60% (**a**), 70% (**b**), 80% (**c**) and 90% (**d**).

## References

[1] Wu M., He M., Hu Q., Wu Q., Sun G., Xie L., Zhang Z., Zhu Z., Zhou A. (2019). Ti_3_C_2_T_x_ MXene-Based Sensors with High Selectivity for NH_3_ Detection at Room Temperature. ACS Sens..

[2] Bhardwaj A., Kumar A., Sim U., Im H., Song S. (2020). Synergistic enhancement in the sensing performance of a mixed-potential NH_3_ sensor using SnO_2_@CuFe_2_O_4_ sensing electrode. Sens. Actuators B Chem..

[3] Meng W., Dai L., Meng W., Zhou H., Li Y., He Z., Wang L. (2017). Mixed-potential type NH_3_ sensor based on TiO_2_ sensing electrode with a phase transformation effect. Sens. Actuators B Chem..

[4] Li H., Lee C., Kim D., Lee J. (2018). Flexible room-temperature NH_3_ sensor for ultrasensitive, selective, and humidity-independent gas detection. ACS Appl. Mater. Interfaces.

[5] Kim S., Koh H., Ren C., Kwon O., Maleski K., Cho S., Anasori B., Kim C., Choi Y., Kim J. (2018). Metallic Ti_3_C_2_T_x_ MXene gas sensors with ultrahigh signal-to-noise ratio. ACS Nano.

[6] Naderi H., Hajati S., Ghaedi M., Dashtian K., Sabzehmeidani M. (2020). Sensitive, selective and rapid ammonia-sensing by gold nanoparticle-sensitized V_2_O_5_/CuWO_4_ heterojunctions for exhaled breath analysis. Appl. Surf. Sci..

[7] Vardhan D., Devi C., Nagaraju P., Muralikrishna P., Kumar B., Upender G. (2024). Room temperature sensing of ammonia and formaldehyde gases through novel anisotype heterojunction of p-Co_3_O_4_/n-Gd_0.1_Ce_0.9_O_2_-δ as highly responsive and stable sensors. Mater. Chem. Phys..

[8] Atkare S., Kaushik S., Jagtap S., Rout C.S. (2023). Room-temperature chemiresistive ammonia sensors based on 2D MXenes and their hybrids: Recent developments and future prospects. Dalton Trans..

[9] Wu R., Guo S., Li Y., Qi M., Ge B., Song J. (2024). Improving the sensing performance of rambutan-like W_18_O_49_ based gas sensor for n-butanol by Ni doping. Sens. Actuators B Chem..

[10] Brophy R., Junker B., Fakhri E., Arnason H., Svavarsson H., Weimar U., Bârsan N., Manolescu A. (2024). Ultra Responsive NO_2_ silicon nanowires gas sensor. Sens. Actuators B Chem..

[11] Hjiri M., Algessair S., Dhahri R., Albargi H., Ben Mansour N., Assadi A., Neri G. (2024). Ammonia gas sensors based on undoped and Ca-doped ZnO nanoparticles. RSC Adv..

[12] Liu Y., Shi T., Chen T., He W., Chen M., Cao D. (2019). The naked-eye NH_3_ sensor based on fluorinated graphene. Sens. Actuators B Chem..

[13] Lorencova L., Bertok T., Filip J., Jerigova M., Velic D., Kasak P., Mahmoud K., Tkac J. (2018). Highly stable Ti_3_C_2_T_x_ (MXene)/Pt nanoparticles-modified glassy carbon electrode for H_2_O_2_ and small molecules sensing applications. Sens. Actuators B Chem..

[14] Qin Q., Olimov D., Yin L. (2022). Semiconductor-Type Gas Sensors Based on γ-Fe_2_O_3_ Nanoparticles and Its Derivatives in Conjunction with SnO_2_ and Graphene. Chemosensors.

[15] Ma Y., Yue Y., Zhang H., Cheng F., Zhao W., Rao J., Luo S., Wang J., Jiang X., Liu Z. (2018). 3D synergistical Mxene/reduced grapheme oxide aerogel for a piezoresistive sensor. ACS Nano.

[16] Yin Y., Liu C., Wang F., Yang L., Han A., Gao J. (2022). The applicantion progress of two-dimensional Mxene material in the field of gas sensor. J. Funct. Mater..

[17] Liu X., Ma T., Pinna N., Zhang J. (2017). Two-dimensional nanostructured materials for gas sensing. Adv. Funct. Mater..

[18] Naguib M., Kurtoglu M., Presser V., Lu J., Niu J., Heon M., Hultman L., Gogotsi Y., Barsoum M. (2011). Two-dimensional nanocrystals produced by exfoliation of Ti_3_AlC_2_. Adv. Mater..

[19] Lu L., Zhang C., Zou Y., Xu F., Sun L., Xiang C. (2024). Room-temperature humidity-resistant highly sensitive ammonia sensor based on a porous MXene/Na_2_Ti_3_O_7_ @polyaniline composite. Sens. Actuators B Chem..

[20] Wang T., Zhu L., Kanda H. (2023). Ti_3_C_2_ MXene-TiO_2_ hybrid-modified U-bend fiberoptic sensor for improved refractive index sensitivity and ammonia detection. Sens. Actuators B Chem..

[21] Yu H., Dai L., Liu Y., Zhou Y., Fan P., Luo J., Zhong A. (2023). Ti_3_C_2_T_x_ MXene-SnO_2_ nanocomposite for superior room temperature ammonia gas sensor. J. Alloys Compd..

[22] Hou M., Jiang G., Guo S., Gao J., Shen Z., Wang Z., Ye X., Yang L., Du Q., Yi J. (2023). Mxene Ti_3_C_2_T_x_ derived lamellar Ti_3_C_2_T_x_-TiO_2_-CuO heterojunction: Significantly improved ammonia sensor performance. Arab. J. Chem..

[23] Yuan G., Wang D. (2017). A piezoelectric six-DOF vibration energy harvester based on parallel mechanism: Dynamic modeling, simulation, and experiment. Smart Mater. Struct..

[24] You J., Wang L., Xi F., Shen J. (2020). Decoupling algorithm and maximum operation frequency of a novel parallel type six-axis accelerometer. IEEE Sens. J..

[25] Hassanein S., Ali R. (2023). Investigation of the Morphological, Optical, and D.C Electrical Characteristics of Synthesized (Bi_2_O_3_/ZnO) Nanocomposites, as Well as Their Potential Use in Hydrogen Sulfide Gas Sensor. Trans. Electr. Electron. Mater..

[26] Wang C., Niu Q., Liu D., Dong X., You T. (2023). Electrochemical sensor based on Bi/Bi_2_O_3_ doped porous carbon composite derived from Bi-MOFs for Pb^2+^ sensitive detection. Talanta.

[27] Chang C., Xue Q., Wang R., Liu Z., Liu Y., He L., Liu F., Xie H. (2023). Development of a novel sensor based on Bi_2_O_3_ and carbonized UIO-66-NH_2_ nanocomposite for efficient detection of Pb(II) ion in water environment. Appl. Surf. Sci..

[28] Hieu N., Van Phuoc C., Hung N., Anh C., Phan A., Nah J., Jeong J., Huy P., Kim D. (2023). A highly stable humidity sensor based on a new Bi_2_O_3_/CNT hybrid nanostructure. Sens. Actuators A Phys..

[29] Pandeeswari R., Sonia T., Balamurugan D., Jeyaprakash B. (2022). Highly Selective Dimethylamine Vapour Sensors Based on Spray Deposited β-Bi_2_O_3_ Nanospheres at Low Temperature. Sens. Imaging.

[30] Alhabeb M., Maleski K., Anasori B., Lelyukh P., Clark L., Sin S., Gogotsi Y. (2017). Guidelines for synthesis and processing of two-dimensional titanium carbide (Ti_3_C_2_T_x_ MXene). Chem. Mater..

[31] Kuang D., Guo X., Zhu Z., Ding Y., Sun X., Wu Z., Zhang L., Zhou Y., He Y. (2021). Enhanced room temperature ammonia response of 2D-Ti_3_C_2_T_x_ MXene decorated with Ni(OH)_2_ nanoparticles. Ceram. Int..

[32] Chaudhary N., Singh A., Aswal D., Debnath A., Samanta S., Koiry S., Sharma S., Shah K., Acharya S., Muthe K.P. (2017). Electron beam modified zinc phthalocyanine thin films for radiation dosimeter application. Synth. Met..

