# Supplementary material for: High Sensitivity Bi2O3/Ti3C2Tx Ammonia Sensor Based on Improved Synthetic MXene Method at Room Temperature
Source: Sensors (Basel). 2024 Oct 10;24(20):6514. doi: 10.3390/s24206514 (PMC11510815; doi:10.3390/s24206514)
Supplement: Supplementary file 1 [file sensors-24-06514-s001.zip › sensors-3204337-supplementary.pdf]

Supplementary Information

Supplementary Figure 1, as supplementary information to Figure 8 in the manuscript, page 8, shows the response of BO/M-5 sensor to detect 100 ppm of acetone, ethanol, DMF, formaldehyde, acetic acid and methanol gas at room temperature.

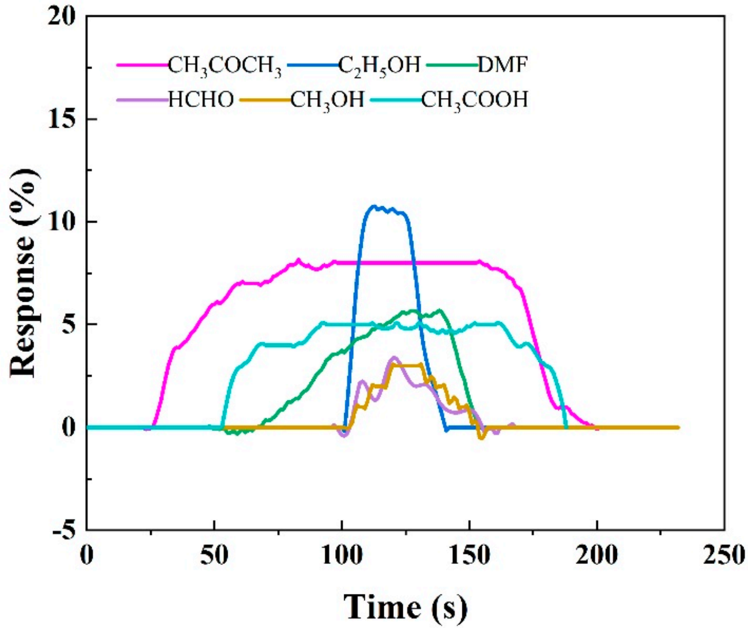

Supplementary Figure S1. The response of BO/M-5 sensor to detect 100 ppm of acetone, ethanol, DMF, formaldehyde, acetic acid and methanol gas

Supplementary Figure 2, as supplementary information to Figure 9 in the manuscript, page 8, shows the response of BO/M-5 gas sensor to 100 ppm of ammonia at relative humidity 60% (a), 70% (b), 80% (c) and 90% (d).

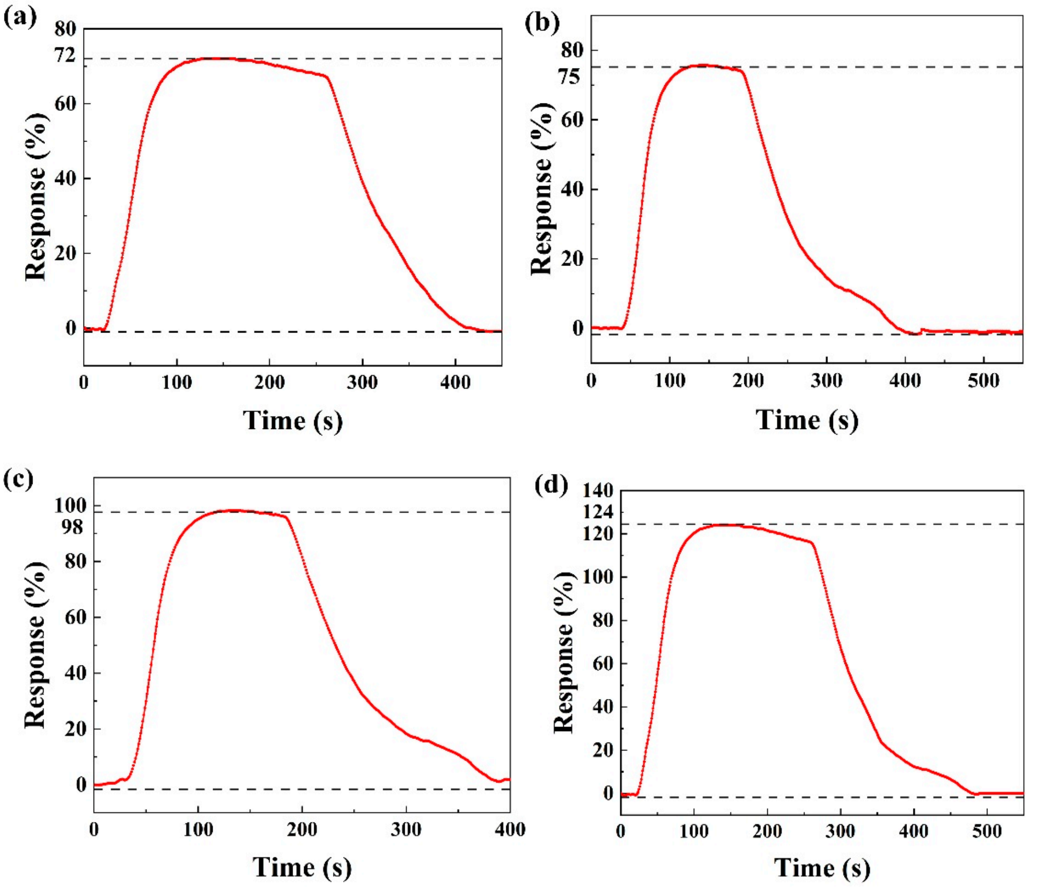

Supplementary Figure S2. The response of BO/M-5 gas sensor to 100 ppm of ammonia at relative humidity 60% (**a**), 70% (**b**), 80% (**c**) and 90% (**d**)
